# Effects of Opioids on Immune and Endocrine Function in Patients with Cancer Pain

**DOI:** 10.1007/s11864-023-01091-2

**Published:** 2023-05-05

**Authors:** Anna Bradley, Jason W Boland

**Affiliations:** 1 Charing Cross Hospital, Imperial College Healthcare NHS Trust, Fulham Palace Road, London, W6 8RF UK; 2 Wolfson Palliative Care Research Centre, Hull York Medical School, University of Hull, Hull, HU6 7RX UK; 3 Hull York Medical School, University of York, York, YO10 5DD UK

**Keywords:** Opioids, Cancer, Immune, Endocrine, Treatment, Pain

## Abstract

Opioids are an important treatment in managing cancer pain. Uncontrolled pain can be detrimental to function and quality of life. Common adverse effects of opioids such as sedation, constipation and nausea are well recognised, but opioid effects on the endocrine and immune systems are less apparent. The evidence for the immunomodulatory effects of opioids suggest that some opioids might be immunosuppressive and that their use might be associated with reduced survival and increased rates of infection in patients with cancer. However, the quality of this evidence is limited. Opioid-induced endocrinopathies, in particular opioid-induced hypogonadism, may also impact cancer survival and impair quality of life. But again, evidence in patients with cancer is limited, especially with regard to their management. There are some data that different opioids influence immune and endocrine function with varying outcomes. For example, some opioids, such as tramadol and buprenorphine, demonstrate immune-sparing qualities when compared to others. However, most of this data is preclinical and without adequate clinical correlation; thus, no opioid can currently be recommended over another in this context. Higher opioid doses might have more effect on immune and endocrine function. Ultimately, it is prudent to use the lowest effective dose to control the cancer pain. Clinical presentations of opioid-induced endocrinopathies should be considered in patients with cancer and assessed for, particularly in long-term opioid users. Hormone replacement therapies may be considered where appropriate with support from endocrinology specialists.

## Introduction

Opioids are often used in the treatment of cancer pain, providing analgesic benefit in approximately 75% of patients [1, 2]. They are no longer prescribed only for patients with cancer approaching the end of life but at earlier stages of disease as well. Therefore, there are ultimately more people with cancer (or its treatment)-related pain taking opioids for longer periods of time. As cancer survival rates continue to improve, there is also a growing group of survivors who remain on opioids after their oncological treatment has been completed [3].

Common opioid adverse effects, such as sedation, confusion, constipation, nausea and drowsiness, are widely acknowledged and assessed for ongoing clinical review [4]. However, there are many less-well known complications of opioids, including effects on the immune and endocrine systems.

The immunomodulatory changes may alter the growth of the malignancy itself, increase the risk of infection and potentially impact the efficacy of immunotherapies [3, 5]. Opioid-induced endocrinopathy is underdiagnosed and therefore undertreated [6]. Opioid effects on the hypothalamic-pituitary-gonadal axis may ultimately lead to poorer response to analgesia and increase mortality [7].

Robust clinical evidence on how the immune and endocrine effects of opioids impact patients with cancer is limited. Much of the data are from preclinical studies or from patients with noncancer pain taking opioids. This article summarises the evidence on the effects of opioids on immune and endocrine function and offer practical recommendations that could be considered in an attempt to manage these effects.

## Immune function in patients with cancer takings opioids

Opioids have been shown in vivo to affect immunity both directly on immune cells and indirectly via immune mediators and the central nervous system [8]. Direct effects are either through immune cells expressing opioid receptors or via opioids binding to non-opioid receptors such as toll-like receptor 4 (TLR-4). Mu-opioid receptor activation can reduce natural killer (NK) cell cytotoxicity [8]. However, the clinical implications of these preclinical studies are unclear. The overall trend suggests that most opioids are immunosuppressive; however, there are conflicting data depending on the population and the opioid studied [3].

Central immunosuppressive effects are either through the periaqueductal grey matter and sympathetic nervous system which in turn releases immunosuppressive amines that suppress NK cell cytotoxicity or through acute morphine binding to D1 receptors which ultimately inhibits splenic NK cell cytotoxicity through the release of neuropeptide Y [8].

**Fig. 1:**
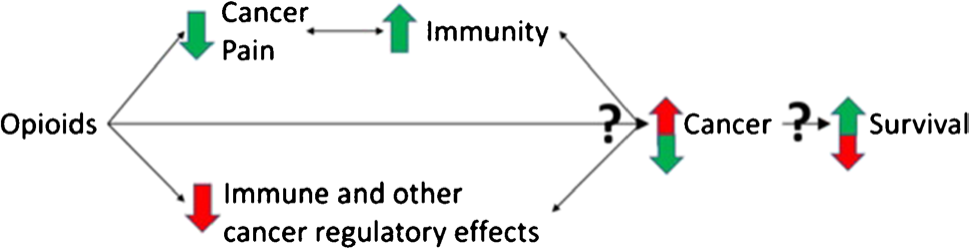
Triangulation of the effects of opioids on pain, immunity and cancer (reproduced from Boland, J.W. Effect of Opioids on Survival in Patients with Cancer. Cancers **2022**, 14, 5720).

Pain itself might also have immunosuppressive effects, through increased release of endogenous opioids, and this has been shown to potentially influence cancer growth in animal models [9•, 10, 11]. Clinical data is lacking. The relationship between pain, opioids, immunity and cancer is summarised in Fig. 1. Clinically, when considering immune function alone, it is a difficult balance between the beneficial use of opioids to manage cancer pain and promote immune function, alongside the known potential immunosuppressive effects of some opioids. However, the numerous other benefits of adequately controlling cancer pain, including on degree of psychological distress and quality of life, must be taken into account during pain management [12].

### Immunomodulatory effects between opioids

The majority of opioid-immune studies refer to morphine, and most data are from preclinical studies [13•]. Of the limited studies on specific opioids other than morphine, there is variation in immune activity between opioids [13•]. This is due to the structure of the different opioid molecules [14]. For example, animal studies of tramadol suggest that it may dose-dependently enhance immunity, perhaps related to its serotonergic effect, and buprenorphine is immune neutral [15, 16]. However, due to a lack of comparative studies studying relevant clinical endpoints, such as survival, based on immune function alone, no one opioid can be recommended over another [10].

### Immunity, opioids and survival in patients with cancer

Survival in patients with cancer is one of the most important hard clinical endpoints. It reflects an interaction of many factors that can be influenced by opioids. Evidence has been emerging over recent years that opioids may impact survival in patients with cancer, although there are differences between studies. A vital role of the immune system in patients with cancer is its ability to control and destroy malignant cells [8]. As well as the general immunosuppressive effects of opioids, they can also have direct effects on cancer cells and their microenvironments by influencing angiogenesis, cancer cell development and apoptosis [9•, 17].

In a systematic review of seven longer-term studies of patients with cancer taking opioids (as opposed to those in patients at the end of life), six indicated that opioids were likely to be associated with a shorter survival. However, none of these was powered to assess the effect of opioids on survival as the primary endpoint. Control groups were also not directly matched [17]. A growing number of more recent observational studies have also suggested that opioid use may decrease survival in patients with cancer [18–22]. A prospective study in adults with advanced, incurable cancer also demonstrated an association between opioid use and decreased survival in a multivariate model. However, the strength of this relationship weakened in a small sub-group when adjusting for CRP, suggesting the hypothesis that the relationship between opioid use and survival in cancer may be in part related to degree of tumour inflammation [23•].

One RCT reported a nonsignificant trend for shorter survival when taking oral opioids compared to intrathecal opioids for pain control (6-month survival 37% versus 54%, *p*=0.06). Pain control was also reported to be better, with less opioid toxicity in the intrathecal group [24]. However, availability of long-term intrathecal opioid delivery systems is limited and potentially not cost-effective or convenient in patients with advanced cancer.

In contrast to the trends seen above, a recent large database study in older adults with advanced pancreatic cancer demonstrated a longer median survival in those prescribed opioids [25]. Hypotheses for why this may be the case included that patients on opioids may be more likely to be receiving more individualised treatment as this group were also more likely to have been referred to palliative care. Studies in patients in the last days or weeks of life (end of life) in a previously discussed systematic review did not find an association between opioid dose and survival. The patient group in the included studies was diverse and spread across multiple settings [17]. Another systematic review of non-surgical patients with cancer did not find any study that showed opioid use impacted clinical outcomes, including survival [10].

A systematic review indicates that pain itself might be an independent negative prognostic factor for overall survival, in 11 out of 17 included studies in patients with advanced prostate cancer, although this association was not seen in other cancers. Detrimental effects of opioids on survival might therefore be partly offset by improvements in pain in certain patient groups (Fig. 1) [26].

There are many confounding factors in the inter-relationship between cancer, pain, opioids and survival. For example, those with more aggressive tumours might have more pain and therefore require opioids [2]. The clinical implications of current research are therefore unclear. Although there may be an association between opioid use and survival, likely due to the well-recognised impact of opioids on the immune system, no causality can be confirmed due to a lack of randomised control trials (RCTs).

### Opioids and infections in patients with cancer

Patients with cancer are at increased risk of infection due to the cancer itself or anti-cancer treatments [27]. Use of opioids may increase this risk further. Chances of infection may be dose related, with increased risk the higher the dose of opioid [28]. The risk for developing infection increased by 2% per 10 mg increase in the daily oral morphine equivalent [29]. A difference in opioids is also reported as people taking morphine were at increased risk compared to those taking oxycodone [30].

### Impact on opioid prescribing

The key points to consider when approaching opioid prescribing and managing the immune effects are summarised in Table 1. Individual patients’ immune response will vary, and there are differences between cancer types [10]. Therefore, the immunomodulatory effect of opioids will not always be predictable. Some studies have indicated that there may be a dose-related effect, with higher doses of opioids causing greater immunosuppression. However, this has also been questioned given the significant limitations in study design with greatly varying doses of opioids (often with very high doses being used even in the ‘lower dose’ groups) and a lack of matched controls [9•]. Despite this, and considering that other opioid side effects improve with dose reduction [34], it would be prudent for clinicians to use the lowest dose of opioid required to adequately control pain. Opioid-sparing strategies to manage pain should also be considered, including interventions such as radiotherapy and nerve blocks and non-opioid analgesia such as non-steroidal anti-inflammatories, antidepressants and anticonvulsants [32, 33]. This is particularly true for patients who are likely to have a longer prognosis and in cancer survivors [11, 31].

**Table 1:** Key points in managing opioid-induced immunomodulatory effects [9•, 13•, 15, 16, 31–33]

1	Consider opioid-sparing strategies, such as disease-modifying interventions, nerve blocks or non-opioid analgesics
2	Different opioids might have varying effects on the immune system. Tramadol and buprenorphine might be more immune-sparing
3	Clinicians should use the lowest opioid dose required to adequately control cancer pain; however, there is limited evidence for a dose-related effect of opioids on the immune system and survival

As more evidence emerges, prescription of opioids that appear to be more immune-sparing, such as tramadol and buprenorphine, could be recommended. Tolerability and varying efficacy in managing cancer pain should also be considered when choosing an appropriate opioid [35]. Ultimately, prescribing should always be individualised to the patient and their specific circumstances.

## Endocrine function in patients on opioids

Opioids have been shown in animal and human studies to affect multiple key hormonal pathways in users, with most studies focussing on the effects of the hypothalamic-pituitary-gonadal axis [3]. However, clinically there is limited awareness of opioid-induced endocrinopathy as a side effect, and it is often overlooked [6]. Symptoms are often non-specific and could be wrongly attributed to the patient’s cancer and its complications or treatment [36]. The clinical implications have also not been systematically evaluated outside of small studies [37].

Very few studies on the endocrine effects of opioids are designed solely in patients with cancer. Studies including noncancer patients taking opioids should be interpreted with caution due to wide variations within the population. Cancers themselves may also significantly alter hormone levels and function [38]. Cancer treatments, including surgery, radiotherapy and chemotherapy, are similarly associated with endocrine effects, sometimes presenting long after treatment has been completed [38].

### Opioid-induced hypogonadism

Opioids inhibit the hypothalamic-pituitary-gonadal axis by acting primarily on mu-opioid receptors in the hypothalamus [3]. This in turn inhibits the secretion of gonadotrophin-releasing hormone, which then decreases the release of luteinising hormone (LH) and follicle-stimulating hormone (FSH) from the pituitary [3]. This ultimately results in reduced testosterone and oestrodiol release by the gonads [3]. Hypogonadism is further precipitated by opioids increasing prolactin secretion by the pituitary gland, which in turn reduces the secretion of testosterone. Suppression of adrenal androgen release and increased peripheral catabolism of testosterone also add to the presentation of opioid-induced hypogonadism [39].

A recent systematic review supported these findings in the cancer population. In five cross-sectional studies examining the effect of long-term opioid therapy (at least 4 weeks) on endocrine biomarkers: lower levels of LH were found compared to control in both sexes, FSH levels were lower compared to control in postmenopausal women (with no change in males), and significantly lower total testosterone and free testosterone were seen in male patients but not postmenopausal females. Oestradiol levels did not change significantly, but this may be due to the inclusion of primarily postmenopausal women [40•].

The prevalence of opioid-induced hypogonadism is between 21 and 86%. The wide range is due to the heterogeneity of study populations (different opioids, different doses, age, impact of other medications and comorbidities) [41]. However, it should also be considered that sexual dysfunction is also common in patients with cancer, both due to the malignancy itself and the oncological treatments used [42]. The prevalence is estimated to be 66% in women with cancer [43].

Despite the high prevalence, androgen deficiency amongst opioid users is underdiagnosed [41]. This may be due to lack of awareness by clinicians or due to the presenting symptoms being attributed to other causes [41]. Symptoms may include fatigue, depression, loss of libido, impotence in males, menstrual cycle disturbance in women and infertility [41]. It has been associated with poor quality of life [44]. Hypogonadism may also be associated with hyperalgesia [7], which is significant in the context of patients taking opioids for cancer pain. Low androgen levels may be linked to an increased incidence of coronary heart disease, possibly due to its influence on cholesterol and lipoprotein levels in the blood [45]. Opioid-induced hypogonadism, as well as direct inhibition of osteoblasts, contributes to increased prevalence of osteopenia and osteoporosis and ultimately increases fracture risk [46].

As with immune function, it may be that the effects of opioids on the hypothalamic-pituitary system have an impact on cancer survival [47]. In one RCT in patients with advanced pancreatic cancer, males who were hypogonadal had a shorter survival than those who were eugonadal; opioid dose was highly correlated to low testosterone [47].

Patients with cancer taking long-term opioids should be routinely screened for symptoms of hypogonadism, and if present, hormonal investigation should be undertaken [48]. In men, this should include serum testosterone, FSH and LH. In women, oestradiol, FSH and LH should be measured. If hormone levels are deranged, other potential influencing factors including medications, comorbidities and hyperprolactinaemia should be considered [49].

### Treatment and clinical implications of opioid-induced hypogonadism

The key points for clinicians to consider when managing opioid-induced hypogonadism are summarised in Table 2. Evidence with regard to management of opioid-induced hypogonadism is limited. This is particularly true for women, as overall more studies have been conducted in men. The degree of hormonal inhibition may depend on the opioid used. Buprenorphine and tapentadol have been shown to cause less suppression of sexual function than methadone and oxycodone/naloxone, respectively [50, 51]. Men taking hydrocodone were also less likely to have hypogonadism than those on fentanyl, methadone or oxycodone in one retrospective cohort study [52].

**Table 2:** Key points in treating opioid-induced hypogonadism [40•, 50–54]

1	Degree of hypogonadism may depend on the opioid used. Buprenorphine, tapentadol and hydrocodone may cause less hypogonadism
2	Risk of hypogonadism is greater with increasing opioid doses
3	Risk of hypogonadism is greater with those taking long-acting over short-acting formulations of opioid
4	Degree of hypogonadism may be greater in those taking long-term opioids

The risk of opioid-induced androgen deficiency is greater with increasing opioid doses and in those taking long-acting opioids compared to those taking regular short-acting doses [40•, 53, 54]. This is due to fluctuations in serum levels of short-acting formulations over the course of the day, compared to a steady state in long-acting medications. The release of hormones is pulsatile such that some release may still happen when serum opioid levels are lower [45]. Length of opioid treatment may be significant in patients with cancer. Individuals taking opioids for 1 year or more had significantly lower sex and gonadotrophic hormone levels compared to those taking them for 1 to 2 months [40•].

Reduction in daily opioid dose may be a treatment option for recognised hormone deficiency. Serum hormone levels return to normal after opioid cessation, even in long-term use. However, no studies have been found that explore the effect of opioid tapering on hypogonadism [55].

If opioid dose reduction, rotation to an alternative opioid or cessation is not feasible, sex hormone supplementation under specialist guidance could be considered to support sexual functioning. However, androgen replacement therapy is not without potential complications. Side effects of testosterone therapy in men could include prostate growth, gynaecomastia, priapism, male pattern baldness, oligospermia, hypercalcaemia, hepatotoxicity (oral formulations, due to first-pass metabolism) and abuse potential [45]. Contraindications include breast or prostate cancer, obstructive sleep apnea, poorly controlled heart failure and haematocrit >50% (risk of polycythaemia) [45]. Serum hormone levels should be monitored alongside LFTs, haematocrit, serum calcium and lipid profile [45]. Women with symptoms of hypogonadism may also benefit from testosterone replacement therapy [56]. Alternative treatment options may include hormone replacement therapy using varying doses of oestrogens and progestins. With these however, there are increased breast cancer and cardiovascular risk [45].

There is little evidence for testosterone replacement therapy in male patients with cancer taking opioids. However, it could improve hypogonadism-induced hyperalgesia [48]. In the noncancer population, an improvement in testosterone levels through supplementation therapy led to improved hypogonadal symptoms but also improvement in pain scores and reduction in the daily morphine equivalent dose [7]. Potential benefits of testosterone replacement therapy were further highlighted in a recent large cohort study of male long-term opioid users with testosterone deficiency who received replacement versus those who did not. All-cause mortality, incidence of cardiovascular events, vertebral or femoral fractures and anaemia were lower after adjusting for covariates in those who received testosterone replacement [57].

### Other endocrine effects of opioids

Opioids may also inhibit the hypothalamic-pituitary-adrenal axis by inhibiting the release of corticotrophin-releasing hormone in the hypothalamus leading to decreased adrenocorticotropic hormone (ACTH) release and reduced serum cortisol levels [3]. Rarely, opioid use has resulted in adrenal crisis through this mechanism in the noncancer population [3]. Risk of adrenal suppression appears greater in those taking higher doses [37]. Other risk factors for opioid-induced adrenal insufficiency are unclear due to the wide heterogeneity of study design [49]. Conflicting findings have been found in one study in patients with advanced cancer. This study in a heterogeneous group demonstrated a correlation between higher opioid doses and higher cortisol levels [58]. This may be due to increased pain (necessitating opioid use) and/or stress causing increased cortisol release [40•].

If a patient shows evidence of adrenal insufficiency, this should be assessed by measuring morning cortisol and an ACTH stimulation test. The high prevalence of glucocorticoid use in the cancer patient population should be considered when measuring cortisol and ACTH [49].

Opioids may also cause increased prolactin levels and therefore lead to gynaecomastia and galactorrhoea. In a recent meta-analysis and systematic review of patients taking opioids for cancer pain, noncancer pain or on maintenance therapy for opioid addiction, hyperprolactinaemia was present in 5 out of 7 studies [59].

No clear effect of opioids on other hormonal pathways has been found, including the somatotropic and the hypothalamic-pituitary-thyroid axes [59].

## Information sharing with patients for informed consent

There is conflicting opinion about how much information regarding the immune and endocrine effects of opioids should be shared with patients in order to give informed consent when commencing opioid therapy [11, 49]. Given that most evidence is from preclinical data and the limited high-quality clinical evidence base in patients with cancer, detailed discussion about immune and endocrine effects when obtaining informed consent for opioid prescription may be premature at this stage [11]. In particular, opioid-immune effects are hard to measure clinically, with many other potential contributing factors [2]. Discussion regarding any potential impact on cancer survival is also likely to impact some patients’ acceptance of an opioid prescription, although this has not been studied [9•].

However, others feel that particularly if a patient is expected to require long-term opioid therapy, they should be informed of the potential risks in order that they may look for the associated symptoms of immune or endocrine dysfunction [49]. This is particularly true of hypogonadism and other endocrinopathies, which are more easily measurable than immune effects and potentially treatable in ways other than opioid dose reduction or opioid switching (options that maybe less appropriate in a patient who has well-controlled cancer pain).

## Future research and next steps

There is a need for prospective studies across a range of clinical settings in order to truly understand the clinical implications of the effect of opioids on the immune and endocrine systems seen in preclinical data. These studies are currently particularly lacking in the cancer population. Future studies should investigate long-term use (with comparisons of continuous versus intermittent use) of opioids in non-surgical cancer pain. Tumour type, pain and survival-related outcomes should be considered [8]. However, it should also be recognised that designing a case-control study in patients with cancer where the control group (i.e. those in pain but not receiving opioids) is matched to the opioid group will be challenging and perhaps unethical [9•]. RCTs with regard to diagnosis of opioid-induced endocrinopathy and the effect of hormone replacement therapy in different cohorts of patients with cancer are needed in order to guide clinical practice and develop evidence-based guidelines (49, 60).

## Conclusions

Careful prescription of opioids, with vigilant monitoring for efficacy and adverse effects, should remain a vital component of a clinician’s approach to managing cancer pain. Relief of suffering is a key objective when delivering patient-centred care. Pain itself is potentially immunosuppressive and in prostate cancer might have a negative impact on survival. Studies generally show an association between opioid use and decreased survival, as well as an increased risk of infection in patients with cancer, partly through their immunomodulatory effects. Weaknesses in the methodology of these studies must be considered.

The impact of opioid-induced endocrinopathies, particularly hypogonadism, is potentially clinically important. These opioid effects might also impact cancer survival and contribute to a worse quality of life for the patient. However, there is a lack of evidence in patients with cancer for the management of endocrinopathies and their clinical implications. Further studies on both the immune and endocrine effects of opioids are needed to ensure that both clinicians and patients can be adequately informed when prescribing or consenting to opioid therapy. In the interim, discussions should be based on the current clinical data. Furthermore, clinicians should not avoid prescribing opioids on the basis of existing evidence on their immune and endocrine effects but should continue to prescribe them as part of a multimodal approach to managing cancer pain.
